# An Overview of Oxidative Stress, Neuroinflammation, and Neurodegenerative Diseases

**DOI:** 10.3390/ijms23115938

**Published:** 2022-05-25

**Authors:** Daniel Mihai Teleanu, Adelina-Gabriela Niculescu, Iulia Ioana Lungu, Crina Ioana Radu, Oana Vladâcenco, Eugenia Roza, Bogdan Costăchescu, Alexandru Mihai Grumezescu, Raluca Ioana Teleanu

**Affiliations:** 1 “Carol Davila” University of Medicine and Pharmacy, 020021 Bucharest, Romania; daniel.teleanu@umfcd.ro (D.M.T.); oana-aurelia.vladacenco@drd.umfcd.ro (O.V.); eugenia.roza@umfcd.ro (E.R.); raluca.teleanu@umfcd.ro (R.I.T.); 2 Department of Science and Engineering of Oxide Materials and Nanomaterials, University Politehnica of Bucharest, 011061 Bucharest, Romania; adelina.niculescu@upb.ro (A.-G.N.); iulia.lunguu@gmail.com (I.I.L.); 3 National Institute of Laser, Plasma and Radiation Physics (NILPRP), 077125 Magurele, Romania; 4 Department of Neurosurgery (I), Bucharest University Emergency Hospital, 050098 Bucharest, Romania; crina.radu95@gmail.com; 5 Department of Pediatric Neurology, “Dr. Victor Gomoiu” Children’s Hospital, 022102 Bucharest, Romania; 6 “Gr. T. Popa” University of Medicine and Pharmacy, 700115 Iasi, Romania; costachescus@gmail.com; 7 “Prof. Dr. N. Oblu” Emergency Clinical Hospital, 700309 Iasi, Romania; 8 ICUB—Research Institute of University of Bucharest, University of Bucharest, 050657 Bucharest, Romania; 9 Academy of Romanian Scientists, Ilfov No. 3, 050044 Bucharest, Romania

**Keywords:** reactive oxidative species, neuroinflammation, neurodegenerative disease, antioxidants

## Abstract

Oxidative stress has been linked with a variety of diseases, being involved in the debut and/or progress of several neurodegenerative disorders. This review intends to summarize some of the findings that correlate the overproduction of reactive oxygen species with the pathophysiology of Alzheimer’s disease, Parkinson’s disease, Huntington’s disease, and amyotrophic lateral sclerosis. Oxidative stress was also noted to modify the inflammatory response. Even though oxidative stress and neuroinflammation are two totally different pathological events, they are linked and affect one another. Nonetheless, there are still several mechanisms that need to be understood regarding the onset and the progress of neurodegenerative diseases in order to develop efficient therapies. As antioxidants are a means to alter oxidative stress and slow down the symptoms of these neurodegenerative diseases, the most common antioxidants, enzymatic as well as non-enzymatic, have been mentioned in this paper as therapeutic options for the discussed disorders.

## 1. Introduction

Neurodegenerative diseases are among the dominant causes of disability and morbidity worldwide, receiving considerable attention due to their high impact on the aging society. These diseases are mainly based on constant deterioration in neuronal function, leading to brain atrophy. Among the most common known neurodegenerative diseases, the following are presented in this review: Alzheimer’s disease (AD), Parkinson’s disease (PD), Huntington’s disease (HD), and amyotrophic lateral sclerosis (ALS) [1,2,3,4,5].

It has been observed that even though different neurodegenerative diseases develop in diverse brain sites and exhibit distinct etiologies, they may act upon similar cellular and molecular processes. Even though there are increasing efforts toward developing appropriate therapies for neurodegenerative diseases, there is still a high demand for efficient agents with therapeutic effects; however, there are still many challenges to face. Even to this day, the exact pathway through which the cellular and molecular mechanisms are involved in the modulation of the evolution of these diseases is unclear. Moreover, the lack of effective biomarkers hinders the possibility of early diagnosis for the majority of these diseases. There is also a need for personalized treatment due to secondary effects, such as inflammation, resulting from the disease’s progression, but most importantly, there is an issue regarding biological barriers. Because the therapeutic agents need to reach the central nervous system (CNS), it is necessary to tailor appropriate vectors that can surpass the brain–blood barrier (BBB) [3,6].

A feature that has been noticed to be common to neurodegenerative diseases is oxidative stress (OS) resulting from the unregulated production of reactive oxygen species (ROS) [7,8]. ROS are known to be linked to several meaningful cellular mechanisms. When ROS are overproduced, they can generate the oxidative deterioration of molecules involved in the progress of aging and several disorders such as cancer and neurodegenerative and cardiovascular diseases. Moreover, ROS-enhanced production may shift the redox balance of the cell towards the oxidative state, leading to its dysfunction and even death. However, naturally, the body has several mechanisms that can counterbalance the outcomes of OS, such as antioxidants. These antioxidants can be either internally produced or provided externally; they are able to detect ROS and reduce the oxidation of the affected cellular molecules. Nonetheless, an impaired antioxidant system may equally contribute to disease pathogenesis [9,10]. In more detail, high oxygen consumption, low antioxidant levels, and low regenerative capacity were observed to induce oxidative damage susceptibility in brain tissues [7].

Therefore, there is a constant need for the development of new approaches that can support the neuroprotective molecules entering the brain, thus implementing more efficient treatments for the disorders of the CNS [11]. In this regard, this paper reviews the significance of oxidative stress in neurodegenerative disorders. There will be a focus on the links between neuroprotection and antioxidants, oxidative stress, neuroinflammation, and mitochondrial dysfunction, as well as the recent advancements in the field.

## 2. Oxidative Stress and Neurodegenerative Events

### 2.1. Reactive Oxygen Species Generated in the Brain

There have been several studies performed in order to determine the role of ROS in the evolution of neurodegenerative diseases, and the results have been promising. Even though results have indicated that ROS do not represent a triggering factor in these diseases, they can probably aggravate disease progression due to oxidative damage as well as their interaction with mitochondria [1,12].

**Figure 1:**
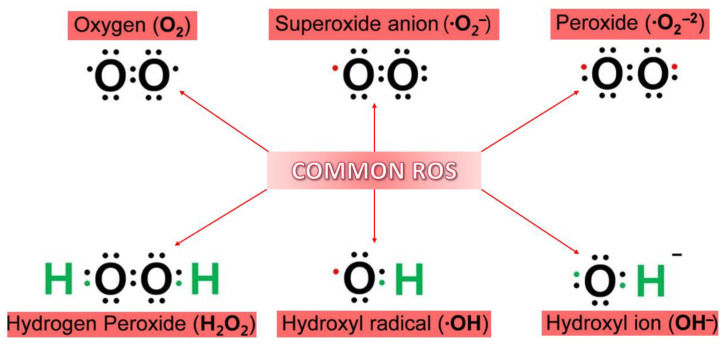
Generally known reactive oxygen species (ROS). Adapted from [13].

Due to its electron structure and its two unpaired electrons, oxygen is prone to radical formation. Therefore, when discussing ROS, they are cataloged as a class of reactive molecules derived from oxygen. This class’s members are known for having short-term life and high reactivity due to their remaining valence electrons. Several ROS can be formed due to the reduction of oxygen through electron addition (Figure 1). Amongst all the types of ROS, ·OH is considered to be the most reactive and the one that can induce a cytotoxic effect [13].

Cellular reactive oxygen species are usually produced from exogenous and endogenous sources. The sources for ROS production exogenously include, for example, ionizing radiation, as well as pharmaceuticals that use ROS production as means of action. Moreover, ROS can also be produced as by-products due to the metabolism of environmental chemicals. On the other hand, the endogenous sources are either mitochondrial or non-mitochondrial ROS-developing enzymes [13,14].

ROS are the result of cellular respiration, a process in which an electron breaks away from the electron transport chain and attaches to oxygen, resulting in superoxide anions (O_2_^−^). The mitochondria take part in the formation of ROS- through the electron transport chain (ETC)-, and indeed around 2% of the oxygen consumed by it is used for this purpose. In normal, healthy cells, up to 90% of ROS are generated as explained. However, the rest are generated by different enzymes. As examples of the enzymes that take part in the production of ROS, the following can be mentioned: monoamine oxidase, dihydroorotate dehydrogenase, and nicotinamide adenine dinucleotide phosphate (NADPH) oxidase (Nox) [13,15].

The above-mentioned ETC is composed of five complexes: Complex I—NADH dehydrogenase, Complex II—succinate dehydrogenase, Complex III—coenzyme Q-cytochrome c reductase, Complex IV—cytochrome C oxidase, and Complex V—adenosine triphosphate (ATP) synthase. The first three complexes are in charge of the production of superoxide anions. Apart from ROS production, Complex I is also responsible for enhancing electron transfers from NADH to coenzyme Q—during which there is another process taking place called proton translocation from the matrix to the intermembrane space. Among the three complexes, Complex II is the one that produces the lowest levels of superoxide anions, but it is also involved in the reduction of coenzyme Q. Complex III is responsible for the production of superoxide anions in the intermembrane space [13].

However, under pathological conditions or within different organs, these enzymes have different capacities for ROS generation. For example, even though Complex I is considered the main source for ROS production in the brain, this is not the case for the heart and lungs, where Complex III is the primary source. Moreover, in the mitochondria, ETC Complex I and Complex III are considered to be the primary generators of ROS. During normal conditions, Complex III generates two times more ROS than Complex I, whereas under disease conditions, Complex I is the main source [13].

Moreover, metalloenzymes present in the organism take advantage of the interaction between oxygen and metal ions for activating molecular oxygen as ROS, the resulting free radicals being intrinsic components of a healthy metabolism. However, as ROS are also toxic, cells have developed complex mechanisms for regulating metal ion interactions and ROS generation. Thus, when regulatory processes break down, the properties that cells utilize for beneficial purposes become destructive [7].

ROS further cause oxidative modifications of major cellular macromolecules, such as lipids, proteins, RNA, and DNA. In complex organisms, lipid peroxidation tends to be more important than DNA oxidation. Protein oxidation is also increasingly recognized as relevant, especially because oxidized proteins may gain a toxic function by forming cytotoxic aggregates [16,17]. 

Therefore, the cumulative effects of different factors (e.g., high oxygen consumption of the brain for high energy needs, elevated levels of polyunsaturated fatty acids in neuronal membranes, high levels of redox transition metal ions, low antioxidant levels, neurotransmitters auto-oxidation) make the brain particularly susceptible to oxidative damage [9,18,19,20].

### 2.2. Parkinson’s Disease

Compared to other neurodegenerative disorders, Parkinson’s disease (PD) is in the top two diseases that affect individuals over 60 years old. Statistics have shown that PD is rare prior to 50 years; amongst the individuals over 65 years old, around 2% are affected, while this percentage is more than double (i.e., 5%) in people over 80 years old [1,13,21,22].

**Figure 2:**
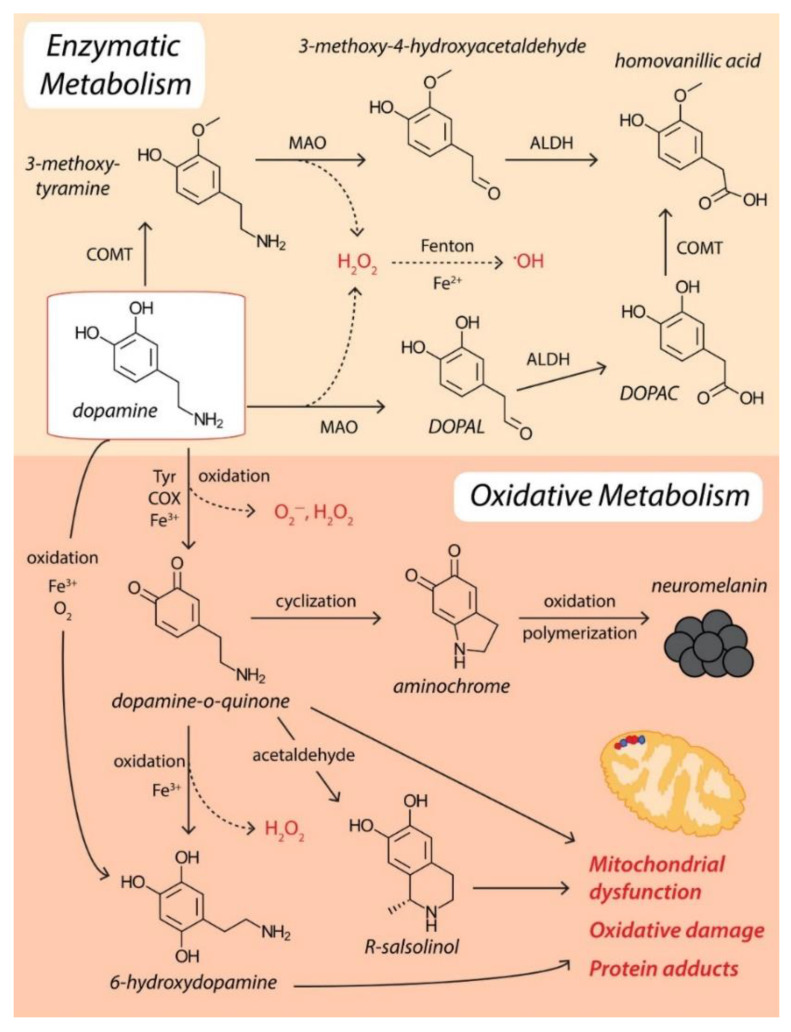
Dopamine metabolism and ROS production. Reprinted from an open-access source [21].

PD is identified when the substantia nigra pars compacta of the brain exhibit dopaminergic neuron loss. This degradation of dopaminergic neurons has been associated with the overproduction of ROS. One of the reasons for the extreme accumulation of ROS can be related to both mitochondrial dysfunctions and inflammation. The main locations in the brain where ROS is generated are mitochondria in the neurons and neuroglia—cells that are not neurons but maintain their protection. The overproduction of ROS is enhanced in this neurodegenerative disorder, and the main reasons are neuroinflammation, mitochondrial dysfunction, age, increased levels of iron and calcium, and dopamine degradation. Moreover, ROS overproduction can be aggravated when there is environmental exposure to pesticides and neurotoxins. Although the exact process that determines dopaminergic neuronal loss is not clear, it has been suggested that ROS is one of the key factors [1,13,23] (Figure 2).

**Figure 3:**
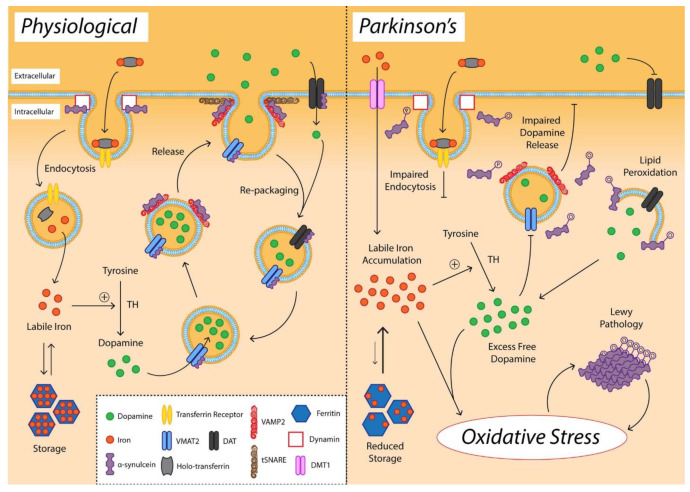
Schematic representation of alterations in dopamine, iron, and alfa-synuclein promoting oxidative stress in the substantia nigra pars compacta. Reprinted from an open-access source [21].

Another characteristic of neurons from the substantia nigra is the age-dependent accumulation of neuromelanin (NM) in cells in the form of large amorphous granules of inconsistent size. NM is a dark brown pigment that accumulates metal ions, coordinates iron, and produces ROS [7,21]. There are two main defense mechanisms that can act against ROS generated by iron–dopamine chemistry, namely, dopamine transporter (DAT) and vesicular monoamine transporter 2 (VMAT2). These neurotransmitter transporters can remove free dopamine from the synapse and pack it into synaptic vesicles to be protected from oxidation. However, with age, nigral expression of DAT gradually declines, indicating an impaired synaptic dopamine clearance. In addition, α-synuclein interacts with VMAT2 during vesicle filling and inhibits DAT-mediated synaptic dopamine reuptake. α-synuclein-VMAT2 interaction also regulates the fusion and clustering of tSNARE-associated vesicles to the presynaptic membrane [21]. For clarity, these processes are visually represented in Figure 3 as a comparison between the healthy and the diseased states.

Furthermore, lipid membranes and cellular proteins can be damaged by ROS production through neurodegeneration, which ultimately builds up OS. The overproduction of free radicals in the respiratory chain is determined by mitochondrial dysfunction. It has been observed that the absence of the mitochondrial Complex I is in direct correlation with PD. Studies have shown that Complex I deficiencies are linked to neural apoptosis, a feature characteristic of PD. This defect has been attributed to the mutation of specific proteins that have the crucial role of preserving the potential of the mitochondrial membrane and having a protective effect against OS. These mutations have been associated with the debut of the disease and can possibly affect the activity of the mitochondria, which leads to the overproduction of ROS and higher exposure to OS [1,23].

Currently, there are no efficient cures for PD; however, the insights provided by understanding ROS mechanisms related to the disease’s evolution can aid the development of treatments for symptom attenuation. There are several neuroprotective approaches that have been determined to decrease mitochondrial oxidative stress. It is known that the adverse effects of free radicals can be diminished by antioxidants and fruits, such as vitamin C and E [1].

According to the information provided on ClincalTrials.gov, a total of 16 studies have previously attempted to correlate PD with oxidative stress. Of the total of these trials, one was withdrawn in 2020 due to the COVID-19 pandemic, seven are registered with unknown status, and eight studies have been completed. Only three studies have public results. The first study (NCT01539837) investigated if the iron chelator deferiprone is well-tolerated, able to chelate iron from various brain regions, and able to ameliorate PD symptomology. The results showed that deferiprone therapy did not adversely affect cognitive function and mood, the evaluated therapy being safe in the short term and decreasing iron in specific brain regions. Nonetheless, future longer-term clinical trials should follow these studies to fully assess the neuroprotective effects of deferiprone [24]. Alternatively, the second study (NCT01470027) focused on checking whether there is a glutathione deficit in the living PD brain that dietary N-acetylcysteine supplementation can mitigate. On a different note, the last study (NCT01467960) evaluated the use of Apolipoprotein D (ApoD) from human serum as a marker of the oxidative stress–inflammation vicious cycle for the early detection of PD.

Moreover, ongoing clinical trials (Table 1) may soon unravel a deeper understanding of ROS mechanisms in PD pathogenesis, offering solutions for its proper management.

### 2.3. Amyotrophic Lateral Sclerosis

Amyotrophic lateral sclerosis (ALS) is also a neurodegenerative disease identified through the loss of motor skills. ALS is considered a rare disease (i.e., 2–3 cases per 100,000 people with European ancestry), being generally manifested at an average age of 50–65 years, with only 5% of cases occurring in individuals 30 years or younger. Nonetheless, it is highly important to better understand and manage this disease due to its severity, as the mean survival from the first symptom is 3–5 years [31].

ALS affects the motor neurons in the nervous system, both upper and lower, which basically means that it affects the whole chain of neurons that project from the cortex all the way to the muscle. For most ALS patients, the cause of the appearance of the disease is unknown. Even though the main symptoms of the patients are related to motor dysfunctions, more than half of patients, during the progress of the disease, show signs of cognitive and behavioral impairment [32].

**Figure 4:**
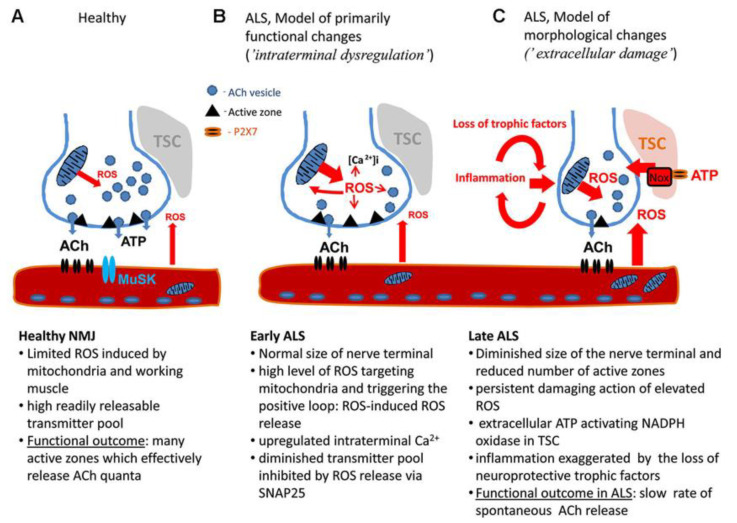
The model of motor nerve terminal dysregulation in ALS. (**A**) Healthy NMJ. (**B**) Pathological changes in NMJ during early stage of ALS. (**C**) Pathological changes in NMJ during late stage of ALS. Abbreviations: Ach—acetyl choline, MuSK—muscle-specific kinase, NMJ—neuromuscular junction, ROS—reactive oxygen species, TSC—terminal Schwann. Reprinted from an open-access source [34].

Due to the fact that there are several factors that can influence the debut and progression of the disease, an etiological investigation has proven to be challenging. Even though one of the first factors that have been identified was OS, its exact role in the progression of the neurodegenerative disease is still not fully understood [33]. A possible explanation has been provided by analyzing ALS mouse models. Thus, it has been reported that nerve terminals are sensitive to ROS, indicating that OS, compromised mitochondria, and increased intracellular Ca^2+^ amplify the presynaptic decline in neuromuscular junctions (NMJ). Further, inflammatory agents and the loss of trophic support are conducive to neurodegeneration [34] (Figure 4).

There are two types of ALS: familial and sporadic; the difference between the two is whether or not there is an inherited genetic element. When discussing sporadic ALS, the element that determines the onset of the disease is unknown. Therefore, it becomes a challenge to determine which causal genes and environmental factors are involved. It has been observed that this type usually develops in patients that are at least 50 years old. On the other hand, in familial ALS, around 20% of the situations, the disorder is a result of mutations in superoxide dismutase-1 (SOD-1). SOD-1 is an antioxidant enzyme, and its main role is to break down reactive oxygen species, such as superoxide, into hydrogen peroxide and molecular oxygen, which are not as toxic to cells. However, hydrogen peroxide, specifically at greater concentrations, is also an oxidant; it can also be decomposed by catalase, which is an enzyme. Within the spinal cord, SOD-1 also has an anti-apoptotic property. On the other hand, mutant SOD-1 creates serious impairments to the respiration and metabolic activities of cells [1,33].

There have been several clinical attempts to try and find appropriate therapies for ALS. A series of relevant clinical trials have been priorly reported connecting ALS with oxidative stress and attempting to combat its various mechanisms of action. More precisely, 16 clinical trials are present on the ClinicalTrials.gov platform: 13 completed studies and 3 with unknown status. However, there is only one drug that has been approved by the FDA and has been shown to inhibit the progression of the disease, namely riluzole. In order to develop effective therapeutic strategies against ALS, the mechanisms underlying the initial pathological events must be identified and addressed. Keeping in mind the role played by OS in this disease, one promising treatment option would be the design of targeted antioxidant therapeutics. Nonetheless, given the complex nature of ALS and the failure of monotherapies, antioxidant treatment should be associated with anti-inflammatory therapeutics for restoring the redox balance [34].

The pathogenesis of this disease is still proving to be challenging, but there is still research in progress regarding the mechanisms that determine the debut as well as the progression of the disorder, OS being a priority [34] (Table 2). 

### 2.4. Alzheimer’s Disease

According to the World Alzheimer Report 2018, around 50 million people were diagnosed with dementia in 2018. Moreover, studies have shown that by the year 2030, there will be 82 million, and the initial number will even triple by the year 2050, accounting for 152 million people living with dementia. Breathtakingly, the report outlines that, globally, there will be a new case of dementia every three seconds [40,41].

Alzheimer’s disease (AD) is defined by a gradual deterioration of cognitive capacities. The degradation of synapses and the death of neurons, especially in the hippocampus—which is the region of the brain that accounts for emotions, learning, spatial orientation, and memory generation—results in brain atrophy. The primary risk factor for the development of AD is age, with disease development affecting almost half the individuals over the age of 85. Moreover, due to the lowering of estrogen levels during menopause and longer life expectancy, women are more likely to develop AD than men [42,43].

Amongst patients that are above 60 years old, AD is one of the most known disabilities worldwide. The pathology of AD is distinguished by an abnormal deposition resulting from aggregated peptides, namely β-amyloid (Aβ), and the build-up of intracellular tau neurofibrillary tangles (NFT). These processes are primarily enhanced and even originated due to OS generation of a disproportion between antioxidants and oxidants, resulting in a rise of oxidants. The reason for this can either be a free radical enhancement or a reduction in the defense of antioxidants [1,44,45].

OS was noticed to assist in AD progression via three main mechanisms that affect cell homeostasis, ROS generation, and the up-regulation of Aβ and p-tau formation. These are macromolecule peroxidation, Aβ metal ion redox potential, and mitochondrial dysfunction [46]. In more detail, the first one represents membrane-associated OS employing lipid peroxidation and production of a neurotoxic aldehyde called 4-hydroxynonenal (HNE). HNE can be detected at the early stages of AD progress, while its levels have been reported to be proportional to the extent of neuronal lesions [47]. Lipid peroxidation may also be involved in the loss of long-term potentiation and other synaptic functions responsible for learning and memory, which are affected in AD brains [48].

In addition, AD brains were reported to exhibit increased neuronal Cu^2+^ and Zn^2+^ levels, up to three times the physiological value of a healthy control brain. These cations can bind the hydrophilic N-terminal ends of Aβ peptides, undergo a redox reaction, and produce considerable amounts of ROS. Thus, there appears to be a positive feedback loop of enhanced oxidation and enhanced ROS production [46]. Moreover, in AD patients, copper is pathologically transferred to senile plaques, causing a deficiency of this metal in the brain cells. This perturbed metal homeostasis is further reflected in the dysregulation of physiological processes governed by metalloenzymes [49].

OS induced by ROS is also considered one of the critical factors in the pathogenesis of AD due to its correlation with the accumulation and deposition of β-amyloid. Even with patients that are in the early stages of the disease, it has been noted that OS can be enhanced due to the build-up of β-amyloid that further results in mitochondrial dysfunction. Studies’ results have shown that the imbalance in OS that is induced by β-amyloid determines a high level of by-products (e.g., protein and DNA/RNA oxidation), as opposed to the levels of antioxidants and their enzymes (e.g., vitamin C) found in Alzheimer’s patients, which are low. Moreover, it has been observed that the imperfections related to the defense mechanisms of antioxidants lead to high levels of OS, which further enhance the production and deposition of β-amyloid [1,13].

**Figure 5:**
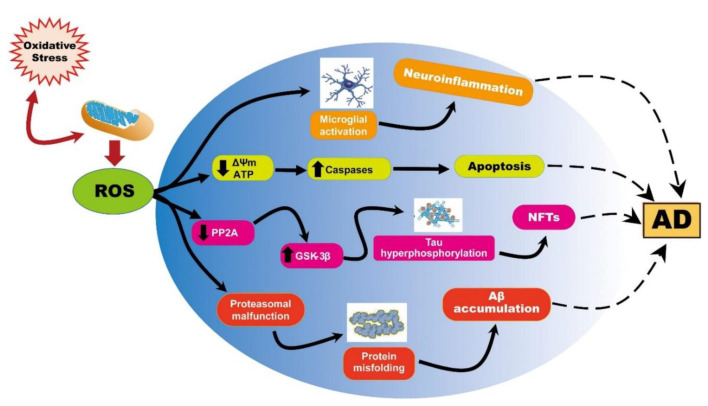
Representation of ROS-induced mitochondrial abnormalities in AD. Reprinted from an open-access source [9].

It has been implied that neuronal disturbance and oxidative imbalance are of critical importance in the inception as well as the advancement of AD. Even though there is still ongoing research trying to understand the functioning mechanism of the excessive production of ROS, it has been observed that in patients suffering from AD, this leads to mitochondrial dysfunction [1,13] (Figure 5). Accumulation of β-amyloid can decrease mitochondrial respiration in neurons and astrocytes by inhibiting Complexes I and IV. Therefore, ROS production can possibly be enhanced through the inhibition of ETC [45].

Research concerning the association of AD with oxidative stress has reached the level of clinical trials, as 17 such studies have been reported on ClinicalTrials.gov. The current status of these trials is as follows: one withdrawn, one unknown, two terminated, and thirteen completed. Results are available for three of these studies, i.e., NCT01388478, NCT00597376, NCT00090402, the latter also being extensively interpreted in an article. In more detail, this study evaluated the effects of supplementation with omega-3 fatty acids alone (ω-3) or omega-3 plus alpha lipoic acid (ω-3 +LA) compared to placebo on oxidative stress biomarkers in AD. Combining ω-3 with LA was reported to slow both cognitive and functional decline in mildly to moderately impaired AD participants, being a safe therapy at the administered doses. However, given that the results were generated from a small sample size (39 participants, out of which 34 completed the 12-month intervention), further evaluation of the combined treatment is needed [50]. 

Increasing research interest in understanding the role of oxidative stress in AD and overcoming its harmful consequences is also evidenced by a series of ongoing clinical trials (Table 3).

### 2.5. Huntington’s Disease

Huntington’s disease (HD) is an inherited neurodegenerative disorder that alters muscle coordination, subsequently leading to mental deterioration and psychological symptoms. The initiation of the disease can occur in individuals as young as 35 years old [59,60].

In order to attain functionality, most proteins have to fold into specific three-dimensional structures. However, there are several conformational states that can be adopted by protein chains. Moreover, the native conformation of the protein chain is usually only partly stable under physiological conditions. This leads to errors occurring in the folding process that can result in misfolded states. These metastable proteins are likely to form aggregates, an action that is linked with cellular toxicity. Furthermore, it is believed that these aggregations could be induced by heritable mutations, as has been observed in HD and the debut of AD and PD [61].

HD is characterized as a protein-misfolding disease, where the Htt protein—a protein called huntingtin produced by the *HTT* gene—causes disruptions in normal biological functions by interacting with other proteins. One of the main problems is that protein misfolding generated by ROS leads to the formation of bodies that agglomerate next to the axons and dendrites of neurons, determining a transmission impediment for the neurotransmitters [59].

The *HTT* gene is made up of a specific DNA segment called CAG (cytosine, adenine, and guanine) trinucleotide. This DNA segment is unstable and expands uncontrollably in the gene. In healthy individuals, the CAG segment is repeated approximately 25 times in the *HTT* gene, whereas individuals with 40–50 repeats develop adult-onset HD, and those with more than 60 CAG repeats tend to manifest HD at a young age [62]. The aggregations generated by mutant HTT (mHTT) create disruptions that could account for the motor and cognitive issues involved in HD patients [1,60]. More specifically, the aggregation process starts with the proteolysis of the misfolded mHTT monomer to produce N-terminal fragments containing a specific sequence of 17 amino acids (N17). N17 plays the role of a nuclear import signal and promotes the formation of aggregates in the nucleus, even though aggregates are also present in the cytoplasm. Nuclear aggregates are further responsible for aberrant transcriptional dysregulation through the sequestration of critical factors that modulate antioxidant genes. Moreover, N17 encodes a specific domain that acts as a ROS sensor for regulating the phosphorylation and location of HTT [63]. 

**Figure 6:**
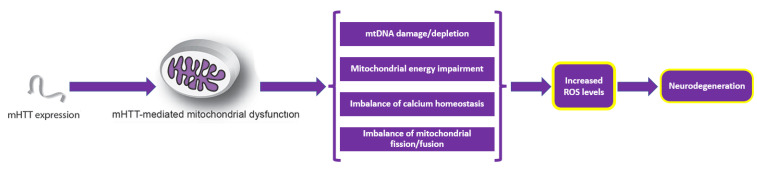
Mutant HTT (mHTT)-induced mitochondria-mediated reactive oxygen species (ROS) accumulation. Adapted from [62].

As mitochondria constitute one of the main sources of ROS and reactive nitrogen species (RNS), their dysfunction has been proposed to play an important role in the pathophysiology of HD. Particularly, mitochondrial dysfunction could lead to overproduction of ROS/RNS and/or failure of the antioxidant defense system. Thus, the redox equilibrium is impaired, leading to decreased physiological cell functions and eventually cell death. Even though abnormal HTT is distributed throughout the entire body, cell degeneration is seen predominantly in the brain, especially within the striatum and cortex [62,64,65,66]. The relationship between mitochondrial dysfunction and neurodegeneration is schematically represented in Figure 6.

Furthermore, studies on animal models and patients with HD revealed the activation of microglia and astrocytes towards producing harmful oxidizing agents. Combined with pro-inflammatory cytokines and other toxic substances, these oxidants can further damage surrounding neurons that were already under strong internal OS caused by mHTT. Concomitantly, the high concentration of reactive species produced by neurons and immune cells activates signaling pathways that maintain pro-inflammatory cytokines and chemokines secretion, thus creating a vicious loop [63]. 

In addition, it has been observed that HD patients exhibit a decreased activity of the Complexes II, III, and IV within the respiratory chain. The restriction of mitochondrial ETC can lead to increased levels of ROS and, concomitantly, lower production rates of ATP [59].

Even though current treatments can diminish the symptoms which are characteristic of these patients, there is still no cure for the disease or even a treatment that can stop its progression [1,60]. It is noticeable that there is a connection between the progression of the disease and the overproduction of ROS and mitochondrial dysfunction; however, it has not been established which one occurs first [1]. Despite strong evidence linking OS and HD, attempts to cure the disease with classic antioxidants have mostly been inefficient [1,67].

An interesting possibility for regulating HD-related neurodegeneration is to use selenium nanoparticles, as reported by Cong et al. [68]. The researchers applied these nanomaterials to transgenic HD models of *Caenorhabditis elegans* and observed that at low dosages, they considerably reduce neuronal death, relieve behavioral dysfunction, and protect the organisms from damage in stress conditions. Moreover, by studying the molecular mechanisms, it was noted that selenium nanoparticles attenuated OS, inhibited HTT aggregation, and downregulated the expression of histone deacetylase family members at mRNA levels. A different perspective on ameliorating motor function and preventing OS in HD individuals is offered by Moghaddam et al. [60]. Specifically, the authors investigated elderberry effects in rat models treated with 3-nitropropionic acid, obtaining promising results. The advantages of elderberry treatment include anti-oxidative and anti-inflammatory effects, a significant reduction in ROS, and a considerable increase in glutathione, which correlated with motor recovery in the tests.

Nonetheless, both these studies perform research on organisms far from humans, and the implications for more complex systems must be studied before considering these therapies efficient for HD patients.

## 3. Neuroinflammation

One of the characteristics of neurodegenerative events is the impairment of the inflammatory response. It has been observed that neuroinflammation together with OS are fundamental aspects that need to be taken into consideration regarding the onset and progression of neurodegenerative disorders, being inextricably linked in their pathogenesis. Inflammatory cells secrete reactive species that produce OS. Some ROS and RNS can further promote intracellular signaling cascades, leading to increased expression of pro-inflammatory genes. Thus, neuroinflammation and OS can stimulate one another, especially in the diseased state. When the body’s reduction and oxidation reactions (redox) are in balance, the inflammatory response acts as a defense mechanism; however, in the case of neurodegenerative events, there is a redox imbalance. Therefore, the inflammatory response does not perform accordingly, generating neuroinflammation in CNS [31,60,69]. 

As a definition, neuroinflammation can be described as the inflammatory response of the CNS to the factors that act against homeostasis. This response includes different types of cells within the CNS, such as astrocytes and microglia. Apart from neurodegenerative diseases, this response can be observed in all types of neurological events, including ischemic, infectious, and traumatic. Since it plays such an important part in the start and progression of these diseases, understanding the processes that go on between the immune system and the CNS is crucial [69].

While it has been noted that, in the case of oxidative stress, the mitochondrial ETC is the primordial root for intracellular ROS, in the case of neuroinflammation, the main inflammatory oxidative enzyme is the over-activated phagocytic NADPH NOX2. It is pivotal to draw attention to the fact that OS and neuroinflammation are two distinct pathological events. Nonetheless, these two events affect and/or cause the other throughout the progress of the disease. Therefore, the inhibition of one of them can result in the suppression of the other [70].

**Figure 7:**
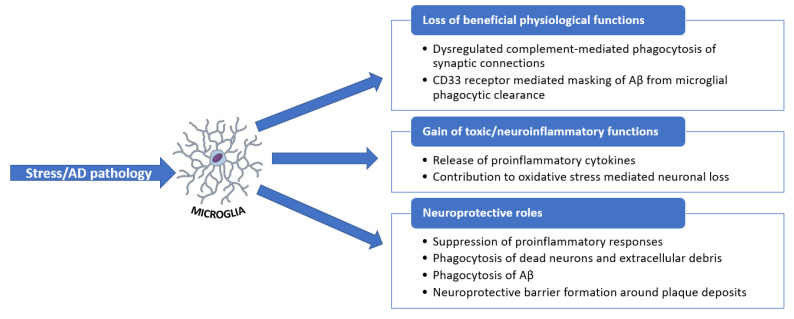
Overview of microglial functions in response to stress and AD pathology. Adapted from [71].

The main element of neuroinflammation is microglia activation. Microglia rapidly respond to imbalances in brain homeostasis occurring due to stress, trauma, disease, or pathology. The activation of microglia results in the discharge of several inflammatory and cytotoxic components responsible for neuroinflammation and neurodegeneration. Nonetheless, microglia also exhibit beneficial roles in maintaining CNS homeostasis and remodeling neuronal circuits. For instance, in the case of AD, microglia offers certain neuroprotection by the phagocytic clearance of Aβ. When microglia lose their beneficial functions, inflammation, synaptic loss, and neuronal damage occur. Additionally, without the clearance of aggregated Aβ, it is noted that the activation of pro-inflammatory signaling pathways promotes inflammation, oxidative stress, and neurodegeneration in AD [70,71]. The roles of microglia in the diseased state are synthesized in Figure 7.

## 4. Neuroprotection and Antioxidants

As mentioned earlier, it has been established that OS has a crucial role in neurodegenerative disorders, being one of the main pathogenic mechanisms underlying neuronal damage and death. It has been observed that antioxidants have to ability to alter OS inside the biological environment and diminish the symptoms of neurodegenerative disorders. Moreover, antioxidant therapies have been noted to prevent, delay, or attenuate the progression of such disorders. Therefore, using antioxidants may provide an interesting option for better managing neurodegenerative diseases [64,72,73].

It is alarming that current statistics show an increased impact of neurodegenerative disorders. There have been numerous studies published regarding the treatment of these diseases through therapeutic approaches. Moreover, although there have been several molecular pathways identified as part of the pathogenesis, OS has been determined to have the most critical role. Because OS can attack multiple molecular pathways that affect neuronal structures, altering OS in the biological environment, either by reducing the overproduction of free radicals or through the defense mechanisms of antioxidants, provides possible therapies for managing neurodegenerative disorders [72,74].

Antioxidant activity can either be provided by a single compound or by an enzyme such as SOD, catalase (CAT), or glutathione peroxide (GPX). Antioxidants can be classified into two distinctive groups: enzymatic and non-enzymatic. The use of antioxidants has gained much attention due to their neuroprotective activity; however, their use in treating neurodegenerative diseases is still debated. Clinical trials have often presented modest effects for antioxidants, while several such substances that were proven effective in animal models did not have comparable activity on disease progression in humans. Moreover, their utilization must be closely monitored, because although they can be beneficial, excessive use can trigger the disease instead of preventing it [72,75,76]. 

### 4.1. Enzymatic Antioxidants

Superoxide dismutase (SOD) is an enzymatic antioxidant, and within the cell, it has been characterized as having the most powerful antioxidant properties. Its activity is mainly protective against ROS. As mentioned earlier, the role of SOD is to break down superoxide anions into less harmful reactive oxygen species. One of the important characteristics of SOD is that it requires a metal ion—most commonly iron, copper, zinc, and manganese—for its activity, which is the reason it is also called a metalloenzyme. However, it is fairly common to exhibit SOD deficiency, since it is greatly correlated with aging: while aging occurs, SOD levels decrease, whereas ROS increases. Therefore, this enzyme is very important to cell health due to the fact that it protects cells from several agents that enhance aging, as well as cell apoptosis. SOD can be naturally found in vegetables, such as broccoli and cabbage, and it has been noted that a controlled supplementation with SOD can protect the immune system, potentially reducing the incidence of diseases [77]. Thus, tackling SOD has been proven a useful tool against various diseases [78,79], including AD [80], PD [81], and ALS [82,83]. 

Catalase (CAT) is an enzymatic antioxidant that can be found in all tissues that require oxygen. It complements SOD in the reduction of H_2_O_2_ to H_2_O and molecular O_2_, and similarly to SOD, it also requires a co-factor to accomplish its activity—usually iron or manganese. This specific antioxidant can be found in high levels in cells, where it constantly searches for H_2_O_2_ molecules. Its efficiency is impressively high, because it can reduce millions of H_2_O_2_ molecules in a one-second time frame. Although it can be abundantly found in cells, it cannot be found in the mitochondria, apart from in rats. The process of hydrogen peroxide molecules degradation in mitochondria is further continued by another enzyme, namely glutathione peroxidase. The lack of this enzyme, or the presence of its mutation, has been observed to be in direct correlation with several neurodegenerative disorders [77]. Catalase treatment was reported to ameliorate memory deficit in AD [84], attenuate neurotoxicity and neuroinflammation [85], and provide significant neuroprotective effects in PD [86].

Glutathione Peroxidase’s (GPx) main activity takes place in the mitochondria. Similar to the other enzymatic antioxidants, it requires a co-factor, in this case, selenium. It has been noted that there are at least eight types of GPx in the human body, denoted from GPx1 to GPx8 -, and that each is linked to a specific chromosome. Therefore, they can be found throughout the body; for example, GPx1 is mostly found in cells, and it is considered to be the most abundant, whereas GPx2 is mainly found in the gastrointestinal tract. Several studies have hypothesized that those patients that exhibit a low activity of the enzymatic antioxidant GPx have a dysfunctional antioxidant protection activity that results in oxidative damage to functional proteins [77].

### 4.2. Non-Enzymatic Antioxidants

Vitamin E is a natural antioxidant derived from plants and can be found abundantly in several dietary products. Its lipid-soluble characteristics allow it to interact with cell membranes, trap ROS, and interfere with the mechanisms of free radical production. Early research studies have shown that the use of vitamin E can reduce the possibility of developing AD and PD as well as reduce the progression of AD. Moreover, the lack of vitamin E in the human body has been linked with the incidence of neurodegenerative disorders [72,87]. Thus, the administration of vitamin E can provide neuroprotective effects by preventing OS in cells and inhibiting apoptosis [88]. In addition to its antioxidant and neuroprotector roles, vitamin E is also involved in anti-inflammatory processes and cell signaling [89]. 

Vitamin C, also known as ascorbic acid, is a notable water-soluble antioxidant and is considered to be the golden standard when it comes to the antioxidant activity expressed. Apart from its antioxidant properties, vitamin C is also known for its involvement in the production of collagen, being a co-factor in the activity of enzymes and the absorption of Fe and being a stimulant agent for the immune system. It has been acknowledged that vitamin C is the most valuable antioxidant agent in the human body. Studies have shown that this antioxidant exhibits neuroprotective properties against neurodegenerative disorders; moreover, it has an essential role in brain protection during ischemic states. Its role against neurodegenerative disorders has been proved due to vitamins C’s ability to neutralize superoxide radicals [72,90]. Vitamin C has particular potential in treating PD, as it can enhance the embryonic midbrain neural stem cells’ differentiation into dopaminergic neurons [88,91].

Coenzyme Q has been classified as a unique antioxidant, which is also lipid-soluble. There are different types of coenzyme Q. However, coenzyme Q_10_ is found in the human body. It is one of the main elements in the mitochondrial ETC, and it can be found in the mitochondrial membrane. Its main role is electron transport to Complex III from Complex I and Complex II. Its antioxidant properties are mainly protective for cell membranes, as well as for plasma lipoproteins. Moreover, this property is specifically essential in the regulation of vitamin C and E levels and preventing hydrogen peroxide-induced apoptosis mediated by ceramide. As mentioned earlier, mitochondrial dysfunction is one factor that contributes to the debut and/or progression of neurodegenerative disorders. In this regard, it has been noted that coenzyme Q can conserve mitochondrial performance and decrease the neuronal deficit in PD. By supplementing coenzyme Q_10_ in patients suffering from PD, which was part of a clinical trial, it has been observed that their functional deterioration could be slowed down [92]. 

Quercetin is another compound recognized for its antioxidant and anti-inflammatory properties that can be used against neurodegenerative diseases [93,94,95,96]. This flavonoid was noticed to protect neurons from oxidative damage, reduce lipid peroxidation, inhibit the fibril formation of amyloid-β protein, and counteract cell lyses and inflammatory cascade pathways, thus being a promising alternative for treating AD [97,98]. Quercetin was also reported to have a cognition-enhancing effect in rat models of PD attributed to its role in decreasing oxidative damage [99]. A recent study also revealed that administration of quercetin can improve mitochondria quality control, reduce OS, increase the levels of the mitophagy markers PINK1 and Parkin, and lower α-synuclein protein expression in 6-hydroxydopamine-lesioned rat models of PD [100].

N-acetylcysteine (NAC) represents an acetylated cysteine compound which has gathered considerable interest as a potential candidate for counteracting neurodegenerative diseases. This substance is a glutathione precursor and exhibits antioxidant and anti-inflammatory properties [101]. Due to their promising activity, NAC-based treatments have reached the level of clinical trials in the fields of AD [54] and PD [25,26]. Moreover, NAC holds promising potential in association with other drugs to create efficient combined therapies for neurodegenerative disorders [101]. 

Essential oils are volatile compounds that occur naturally, being synthesized by plants [102]. During neurodegenerative events, the immune system is weakened, and there is an abnormal overproduction of ROS. Therefore, there is a high requirement for the intervention of free radical scavengers. In this regard, essential oils have gained attention due to their neuroprotective and anti-aging features and their safety and efficient use compared to synthetic drugs. For example, lavender essential oil has exhibited antioxidant as well as anti-apoptotic properties in studies involving rat models [103]. Moreover, many other essential oils from various sources have been investigated in the past decade as antioxidant treatment options for neurodegenerative diseases [104]. For instance, essential oils extracted from *Aloysia citrodora Palau* [105], *Boswellia dalzielii* [106], *Cinnamomum zeylanicum* [107], *Citrus Sinensis* [L.] *Osbeck* [108], *Clinopodium serpyllifolium* (M.Bieb.) Kuntze [109], *Pinus halepensis* [110], *Polygonum hydropiper* L. [111], *Rumex hastatus* D. Don [112], *Salvia officinalis* [113,114], *Stachys* species [115], *Sideritis galatica Bornm*. [116], *Tetraclinis articulata* [117], and *Thymus* species [118,119], have proven promising against AD, whereas *Pulicaria undulata* [120], *Rosa damascene* Mill. and *Lavandula angustifolia* Mill. [121,122] represent important antioxidant sources for treating PD.

## 5. Conclusions and Future Perspectives

In this review, reactive oxidative stress’ complex roles in the most common neurodegenerative diseases, which include Alzheimer’s disease, Parkinson’s disease, Huntington’s disease, and amyotrophic lateral sclerosis, have been discussed. In the past three decades, remarkable efforts have been made in order to diagnose these neurodegenerative diseases from the early stages, which include the identification of biomarkers of neuropathological, biochemical, and genetic nature. At present, it is known that oxidative stress becomes impaired during the process of aging and represents a feature that is expressively involved in the mentioned process, but it is still unclear if it can be used as a marker for early-stage detection of the dysfunction of aging or as an effective therapeutic target [123]. 

As mentioned before, high levels of ROS have been implicated in many neurodegenerative circumstances. Scientists suggest that ROS may be produced through numerous mechanisms and have very complex roles in promoting disease development. In particular, the dysfunction of mitochondria is connected to sustained ROS in neurodegenerative disorders. Even though there has been no substantial evidence presenting their neuroprotective efficacy, research studies have positively attained promising results. Further research is very important in addressing the particular roles of ROS in numerous neurodegenerative conditions and emerging antioxidant-based therapeutic interventions [1]. Biomarkers of ROS could be used as diagnostic instruments or therapeutic targets. Antioxidant therapy, using resveratrol or other nutritional compounds, alongside moderate physical exercise, could positively affect the clinical damage prompted by oxidative stress. Nonetheless, more investigations are required to assess the real efficacy of all these therapeutic interventions [124].

In conclusion, even though multiple studies have been performed to explore the effectiveness of antioxidants in attenuating neurodegenerative symptoms, convincing evidence showing their neuroprotective efficacy is still scarce. However, ongoing clinical trials have the potential to bring more encouraging outcomes, especially when considering antioxidants as adjuvant therapeutic agents alongside other treatments. Further thorough research is paramount in finding the exact roles of ROS in various neurodegenerative conditions and developing antioxidant-based therapeutic interventions. Furthermore, improved knowledge of the mechanisms of mitochondria and oxidative stress in the aging process and neurodegeneration could support new strategies for not only improving quality of life of the elderly but also positively impacting the whole of modern society.

## Figures and Tables

**Table 1:** Summary of active clinical trials investigating Parkinson’s disease in relation to oxidative stress. The studies have been retrieved from clinicaltrials.gov with search keywords “Condition or disease = Parkinson disease” and “Other terms = oxidative stress” and selecting the items with the status “Recruiting” and “Active, not recruiting”.

ClinicalTrials.gov Identifier	Official Title	Intervention/ Treatment	Phase	Estimated Completion Date	Ref.
NCT04459052	Phase II: Physiological Effects of Nutritional Support in Patients With Parkinson’s Disease	Dietary Supplement: N-acetylcysteine Drug: [F-18] Fluorodopa Positron Emission Tomography	Phase 2	1 May 2023	[25]
NCT02445651	Physiological Effects of Nutritional Support in Patients With Parkinson’s Disease	Dietary Supplement: Intravenous and Oral N-acetylcysteine	Not applicable	6 December 2021	[26]
NCT05214287	An N-of-1 Double-blind Randomized Phase 1 Trial of the Safety and Feasibility of (Intermittent) Hypoxia Therapy in Parkinson’s Disease	Drug: Hypoxic Gas Mixture	Phase 1 Phase 2	February 2023	[27]
NCT05110547	Multicenter Study of Blood Biomarkers of Mitochondrial and Peroxisomal Metabolism to Differentiate Idiopathic Parkinson’s Disease From Related Conditions	Biological: Blood Collection	Not applicable	April 2023	[28]
NCT02524405	The Brain Eye Amyloid Memory (BEAM) Study: Validation of Ocular Measures as Potential Biomarkers for Early Detection of Brain Amyloid and Neurodegeneration	Other: Pittsburgh Compound B [11C]-PIB	Not applicable	December 2023	[29]
NCT04491383	Tocotrienols in Parkinson’s Disease (PD): A Pilot, Randomised, Placebo-controlled Trial	Drug: Tocovid Suprabio (HOV-12020) Other: Placebo	Phase 2	December 2024	[30]

**Table 2:** Summary of active clinical trials investigating amyotrophic lateral sclerosis in relation to oxidative stress. The studies have been retrieved from clinicaltrials.gov with search keywords “Condition or disease = Amyotrophic lateral sclerosis” and “Other terms = oxidative stress” and selecting the items with the status “Recruiting” and “Active, not recruiting”.

ClinicalTrials.gov Identifier	Official Title	Intervention/ Treatment	Phase	Estimated Completion Date	Ref.
NCT04788745	Targeting Metabolic Flexibility in ALS (MetFlex); Safety and Tolerability of Trimetazidine for the Treatment of ALS	Drug: Trimetazidine Dihydrochloride	Phase 2	31 March 2023	[35]
NCT04097158	Oxidative Markers and Efficacy in Amyotrophic Lateral Sclerosis (ALS) Phenotypes Treated With Edaravone	Other: Sample Collection	Not applicable	September 2023	[36]
NCT03293069	Conservative Iron Chelation as a Disease-modifying Strategy in Amyotrophic Lateral Sclerosis: Multicentre, Parallel-group, Placebo-controlled, Randomized Clinical Trial of Deferiprone	Drug: Deferiprone Drug: Placebo Oral Tablet	Phase 2 Phase 3	November 2023	[37]
NCT04244630	Mitochondrial Capacity Boost in ALS (MICABO-ALS) Trial	Combination Product: Antioxidants	Phase 2	December 2023	[38]
NCT04259255	Radicava^®^ (Edaravone) Findings in Biomarkers From ALS (REFINE-ALS)	Drug: Edaravone	Not applicable	March 2023	[39]

**Table 3:** Summary of active clinical trials investigating Alzheimer’s disease in relation to oxidative stress. The studies have been retrieved from clinicaltrials.gov with search keywords “Condition or disease = Alzheimer disease” and “Other terms = oxidative stress” and selecting the items with the status “Recruiting” and “Active, not recruiting”.

ClinicalTrials.gov Identifier	Official Title	Intervention/ Treatment	Phase	Estimated Completion Date	Ref.
NCT02800395	Influence of Oxidative Stress and Nutrition Biomarkers on the Cognitive Decline Evolution in Alzheimer Disease	Procedure: Malnutrition Screening and Perioperative Nutritional Support	Not applicable	December 2026	[51]
NCT04430517	Effects of Orally Administered Nicotinamide Riboside on Bioenergetic Metabolism, Oxidative Stress and Cognition in Mild Cognitive Impairment and Mild Alzheimer’s Dementia	Drug: Nicotinamide Riboside	Early Phase 1	30 April 2025	[52]
NCT03514875	Effects of Mitochondrial-targeted Antioxidant on Carotid Artery Endothelial Function and Brain Blood Flow in Mild Cognitive Impairment (MCI) Patients	Dietary Supplement: MitoQ Dietary Supplement: Placebo	Not applicable	1 October 2022	[53]
NCT04740580	Glutathione, Brain Metabolism and Inflammation in Alzheimer’s Disease	Dietary Supplement: Glycine Dietary Supplement: N-acetylcysteine Dietary Supplement: Alanine	Early Phase 1	31 May 2025	[54]
NCT03035851	Aerobic Exercise for Older Adults at Increased Risk of Alzheimer’s Disease and Related Dementias: Harnessing Translational Physiology	Behavioral: Aerobic Exercise Behavioral: Stretch and Strength	Not applicable	January 2025	[55]
NCT02524405	The Brain Eye Amyloid Memory (BEAM) Study: Validation of Ocular Measures as Potential Biomarkers for Early Detection of Brain Amyloid and Neurodegeneration	Other: Pittsburgh Compound B [11C]-PIB	Not applicable	December 2023	[29]
NCT04213391	Randomized, Double-blind, Placebo-controlled, Efficacy and Safety Study of Sulforaphane in Patients With Prodromal to Mild Alzheimer’s Disease	Dietary Supplement: sulforaphane Dietary Supplement: Placebo	Not applicable	1 December 2022	[56]
NCT05145881	Evaluation of Clinical Effect of Probiotics in Alzheimer’s Disease: a Randomized, Double-blind Clinical Trial	Dietary Supplement: Low-dose Probiotics Dietary Supplement: Normal-dose Probiotics	Not applicable	30 June 2023	[57]
NCT05007353	The SINgapore GERiatric Intervention Study to Reduce Cognitive Decline and Physical Frailty (SINGER) Study, Biomarker and Health Service Research Analyses	Behavioral: Structured Lifestyle Intervention Behavioral: Self-Guided Intervention	Not applicable	31 January 2026	[58]

## Data Availability

Not applicable.
